# Treatment sequences and survival outcomes in advanced HR + HER2- breast cancer patients: a real-world cohort

**DOI:** 10.1007/s10549-024-07542-0

**Published:** 2024-11-07

**Authors:** Cornelia A. M. Almekinders, Lishi Lin, Jos H. Beijnen, Gabe S. Sonke, Alwin D. R. Huitema, Vincent O. Dezentjé

**Affiliations:** 1 Department of Medical Oncology, The Netherlands Cancer Institute—Antoni van Leeuwenhoek Hospital, Plesmanlaan 121, 1066 CX Amsterdam, The Netherlands; 2 Department of Pharmacy and Pharmacology, The Netherlands Cancer Institute—Antoni van Leeuwenhoek Hospital, Amsterdam, The Netherlands; 3 Utrecht University, Utrecht, The Netherlands; 4 University of Amsterdam, Amsterdam, The Netherlands; 5 Department of Pharmacology, Princess Máxima Center for Pediatric Oncology, Utrecht, The Netherlands; 6 Department of Clinical Pharmacy, University Medical Center Utrecht, Utrecht University, Utrecht, The Netherlands

**Keywords:** Breast cancer, Endocrine therapy, Real-world, Treatment sequences

## Abstract

**Purpose:**

Palliative treatment options for HR + HER2- advanced breast cancer (ABC) patients have increased, but data is lacking about the optimal treatment sequence. We used real-world data from a comprehensive cancer center to describe applied treatment sequences and we determined treatment-related and survival outcomes.

**Methods:**

Patients aged 18 years and older with HR + HER2- ABC treated with systemic treatment were included in this historic cohort study. Sequential treatment schedules, time to treatment discontinuation, time to chemotherapy, and overall survival (OS) were determined, stratified by first-line treatment.

**Results:**

202 patients were included. They received a total of 650 treatment lines (median 3; range: 1–11). 91 (45%), 25 (12%), 24 (12%), 28 (14%), 22 (11%) and 12 (6%) patients started first-line treatment with non-steroidal aromatase inhibitors (NSAI), NSAI + cyclin dependent kinase 4/6-inhibitors (CDK4/6i), fulvestrant + CDK4/6i, tamoxifen, chemotherapy and other treatment, respectively. 10, 13, and 14 different treatment regimens were given in first, second and third-line, respectively. Of the patients who started first-line NSAI monotherapy (*n* = 91), 3 (3%) died before receiving second-line treatment.

**Conclusion:**

In this real-world cohort, we observed a wide variety of different treatment sequences applied in daily clinical practice, some of which were in discordance with the current guidelines. Fear that patients may never get around to treatment with CDK4/6i if a patient did not start with a CDK4/6i was not supported by our study results.

**Supplementary Information:**

The online version contains supplementary material available at 10.1007/s10549-024-07542-0.

## Introduction

Overall survival (OS) for patients with advanced or metastatic breast cancer (ABC) has improved over the past 20 years. However, median OS is still at less than four years in a large French subcohort with HR + HER2- ABC patients younger than 70 years [1]. Most ABC patients are treated with palliative systemic therapy until unacceptable toxicity, progressive disease, or death occurs. The aim of palliative systemic therapy is twofold: to prolong life and to improve the quality of life.

Endocrine therapy (ET) is the backbone of first and second-line systemic therapy for HR + HER2- ABC patients, with aromatase inhibitors (AIs) as first-line therapy, and fulvestrant as second-line therapy [2]. In the past decade, several new treatment options for HR + HER2- ABC patients became available, such as cyclin-dependent kinase 4/6-inhibitors (CDK4/6i), as well as inhibitors of the phosphoinositide-3-kinase (PI3K)-AKT-mTOR pathway (PI3Ki). Both CDK4/6i and PI3Ki must be combined with ET.

Novel therapy options become available after phase 3 trials, in which the superiority of one (combination of) treatment over another is demonstrated in terms of progression-free survival (PFS) benefit during one treatment line. Effective new treatments often move from later to earlier lines by demonstrating PFS benefit in each separate line. Although a new treatment may be equally effective in a later line in terms of OS benefit, treating as early as possible with the most effective treatment based on PFS benefit is a widely accepted treatment strategy. Empiric data on the best treatment sequence is often lacking and the fear of withholding an effective treatment leads to adaptations of this strategy, even if it comes with added toxicity and costs.

This is for example true for CDK4/6i. Although CDK4/6i were studied extensively in the PALOMA, MONALEESA, and MONARCH studies, all of which have shown a clear PFS-benefit of adding CDK4/6i to ET [3–5], these studies failed to compare placement of CDK4/6i in first-line versus CDK4/6i in second-line treatment head-to-head. This head-to-head comparison is done in the ongoing SONIA-trial (NCT03425838). In the primary analysis, no significant difference was observed in PFS in the first two treatment lines combined (PFS-2). When CDK4/6i were added to second-line ET, less adverse events were observed and costs of treatment were lower than when CDK4/6i were added to first-line ET [6]. This example demonstrates the importance of researching the optimal sequence of novel therapies.

A similar question arises regarding the choice of (backbone) ET. Several studies have shown better results of treatment with AIs than with tamoxifen in first-line [7]. AIs have thus become the preferred treatment in first-line over tamoxifen in ABC setting. However, little is known about the chance of response when tamoxifen is given as first-line therapy, then followed by other (backbone) ET. In the randomized PARSIFAL trial no superiority was shown for fulvestrant over letrozole as backbone ET [8].

Altogether, this underlines the crucial importance of sequencing studies so that patients benefit at maximum of all the available treatment options, but funding is often lacking and sequence studies are not feasible in every situation. When randomized trials are lacking, analysis of real-world data can give useful information on sequence strategies and its effect on OS and time to chemotherapy (TTC) as a proxy for quality of life. Therefore, we described applied treatment sequences for HR + HER2- ABC patients at a comprehensive cancer center. In addition, we determined several treatment-related and survival outcomes of patients treated with different treatment sequences.

## Methods

### Patient selection and data collection

This historic cohort study was conducted at the Netherlands Cancer Institute (NKI), Amsterdam, The Netherlands. All HR + HER2- ABC patients aged 18 years and older who were diagnosed between January 2014 and January 2023 and received palliative treatment were included in this study. The initial search was composed of ‘HR + breast cancer’ as registered diagnosis, combined with any relevant diagnosis treatment combinations (DBCs). These DBCs are standard packages to simplify treatment reimbursement usually given to a group of patients with the same diagnosis. DBCs cover ‘the standard journey’ of ‘the standard patient’ with a certain diagnosis [9]. Only patients who consented to their data being used for research were included in this initial search. After the initial search, patients were screened for eligibility in this study. Patients without HR + HER2- ABC or patients receiving treatment for other tumors than breast cancer were excluded, as were patients participating in double-blinded studies and patients unwilling to receive any type of systemic treatment.

Data on patient characteristics, tumor characteristics and treatment lines were extracted from the electronic medical records Hix (ChipSoft, Amsterdam, The Netherlands). For determination of endocrine resistance at the time of diagnosis of mBC, the definitions of endocrine resistance as stated in the ABC-5-guideline were applied [2]. Data cutoff was the first of August 2023. The conduct of this study was approved by the Investigational Review Board of the NKI and the need for a study-specific written informed consent was waived.

### Outcomes

Treatment outcomes of interest were the number of different treatment lines and the treatment sequence received by patients including the reason of treatment discontinuation. In addition, time to treatment discontinuation (TTD) for first-line treatments, total time on treatment until first chemotherapy regimen (TTC) for all patients starting with first-line ET and OS were determined, stratified by first-line treatment.

When one agent in combination therapies, such as fulvestrant + CDK4/6i, was temporarily discontinued due to toxicity, intercurrent events, or intercurrent treatments, the treatment was classified as combination therapy if the combination was given > 50% of the time. If only one of the agents was given > 50% of the time, the treatment was classified as single-agent therapy.

### Statistical analysis

Descriptive statistics were reported using frequencies for categorical data and means or medians for continuous data depending on the distribution. The treatment regimens received by patients were visualized using Sankey plots. TTD and OS were estimated using the Kaplan–Meier method. The log-rank test was used to compare TTD and OS between different first-line treatments. Groups with five or less patients within a first-line treatment group were excluded from the analyses. This resulted in five different first-line treatment groups. First-line treatment with non-steroidal aromatase inhibitors (NSAI) was used as the reference group for further statistical analyses (based on the SONIA trial). A Bonferroni correction was applied to correct for multiple testing. Thus, an adjusted *p*-value < 0.0125 was considered statistically significant as four comparisons were made for TTD as well as for OS. The cumulative incidence for TTC were determined using the competing risk approach, in which death was regarded as a competing outcome. Cumulative incidences were compared using the Gray’s test, with an adjusted *p*-value < 0.015 considered statistically significant as three comparisons were made [10]. All analyses were performed in R version 4.3.1 [11].

## Results

### Patients

**Fig. 1:**
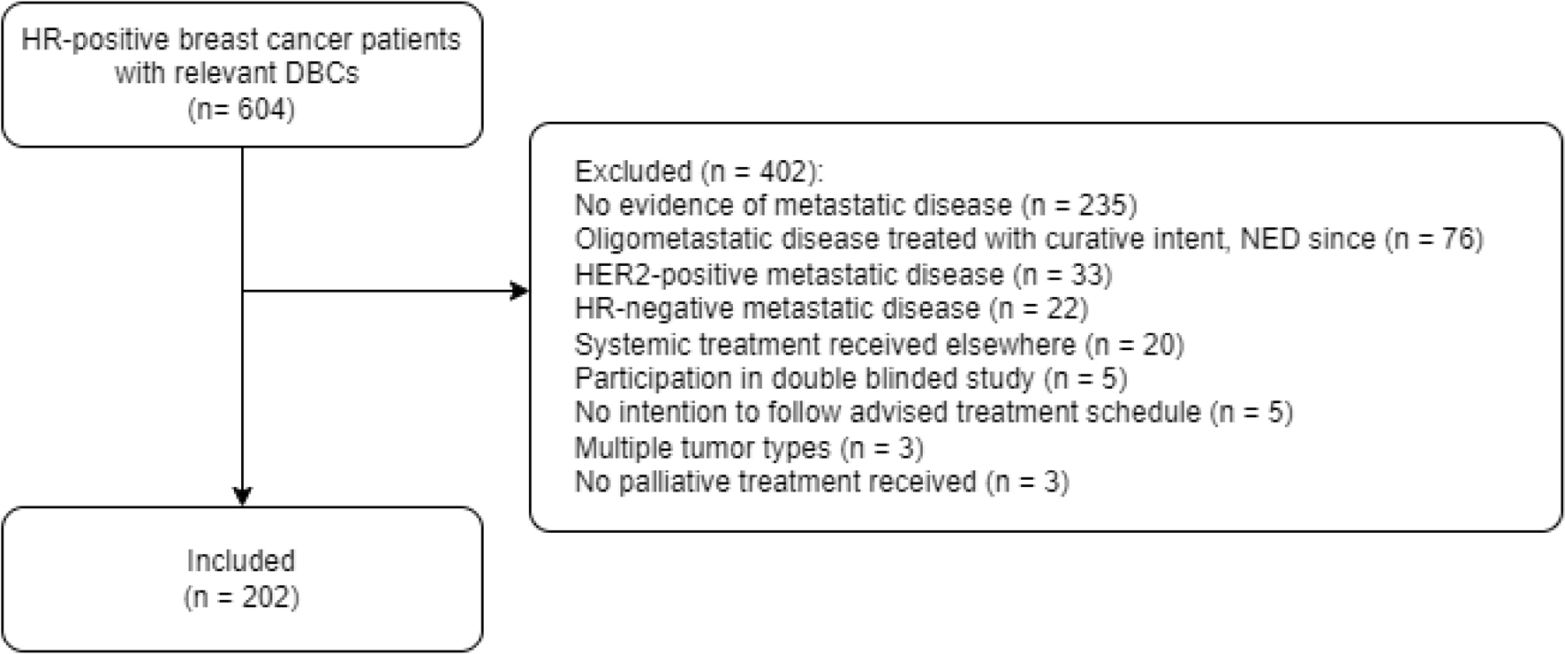
Flow chart of patient selection. *DBCs* diagnosis treatment combinations, *NED* no evidence of disease

Six hundred four patients were identified in the initial selection, of which 402 patients were excluded. The main reasons for exclusion were no evidence of metastastic disease, oligometastatic disease treated with curative intent with no evidence of disease thereafter, and HER2 + ABC (Fig. 1). The final cohort consisted of 202 patients, of which 200 were female. Fifty five percent of patients received any type of systemic treatment in the (neo) adjuvant setting. Patient characteristics at the start of first-line treatment are depicted in Table 1. Patients in the fulvestrant + CDK4/6i group were relatively older and had higher rates of endocrine resistance. Endocrine resistance was lowest in the NSAI and tamoxifen groups. Patients in the tamoxifen group were somewhat younger than patients in other groups.

**Table 1:** Patient characteristics at the start of the first-line treatment

	NSAI	NSAI + CDK4/6i	Fulvestrant + CDK4/6i	Tamoxifen	Chemotherapy	Other*
	*N* = 91 (%)	*N* = 25 (%)	*N* = 24 (%)	*N* = 28 (%)	*N* = 22 (%)	*N* = 12 (%)
Age, years						
Median (IQR)	59 (51–68)	50 (42–58)	66 (59–69)	48 (43–60)	52 (40–58)	59 (50–68)
Age, years						
< 45	9 (10)	8 (32)	1 (4)	11 (39)	7 (32)	2 (17)
45–55	26 (28)	9 (36)	4 (17)	8 (29)	8 (36)	2 (17)
> 55	56 (62)	8 (32)	19 (79)	9 (32)	7 (32)	8 (66)
Sex, female	90 (99)	25 (100)	24 (100)	27 (96)	22 (100	12 (100)
Site of metastatic disease at diagnosis						
Primary tumor + lymph nodes	–	–	–	2 (7)	–	–
Bone only	11 (12)	2 (8)	5 (21)	4 (14)	1 (5)	1 (8)
Visceral	41 (45)	17 (68)	13 (54)	12 (43)	16 (72)	8 (67)
Non bone only, non-visceral	39 (43)	6 (24)	6 (25)	10 (36)	5 (23)	3 (25)
WHO performance status						
0	39 (43)	10 (40)	6 (25)	11 (39)	10 (45)	3 (25)
1	21 (23)	9 (6)	11 (46)	3 (11)	5 (23)	2 (17)
2	6 (7)	1 (4)	1 (4)	2 (7)	2 (9)	–
3	1 (1)	–	–	–	–	–
NA	24 (26)	5 (20)	6 (25)	12 (43)	5 (23)	7 (58)
No. treatment lines Median (range)	3 (1–11)	2 (1–9)	2 (1–5)	4 (1–10)	4 (1–8)	2 (1–5)
Systemic treatment given in (neo)adjuvant setting						
Chemotherapy	33 (36)	11 (44)	19 (79)	8 (29)	14 (64)	7 (58)
Hormonal therapy	34 (37)	13 (52)	24 (100)	10 (36)	14 (64)	8 (67)
HER2i	1 (1)	–	–	1 (4)	2 (9)	–
None	51 (56)	12 (48)	–	17 (61)	7 (32)	4 (33)
Endocrine resistance**						
None	70 (77)	14 (56)	1 (4)	22 (79)	9 (41)	6 (50)
Primary resistance	9 (10)	3 (12)	8 (33)	2 (7)	6 (27)	2 (17)
Secondary resistance	12(13)	8 (32)	15 (63)	4 (14)	7 (32)	4 (33)

*Fulvestrant, exemestane, chemotherapy + endocrine therapy or VEGF inhibitor or experimental treatments

**In accordance with ABC5 guideline: Primary resistance was defined as recurrence of disease < 2 years after start of any adjuvant endocrine therapy. Secondary resistance was defined as new evidence of disease > 2 years after start of any adjuvant endocrine therapy, provided the therapy was continued until the last year before the diagnosis of mBC

CDK4/6i, cyclin-dependent kinase 4/6 inhibitor; IQR, interquartile range; HER2i, human epidermal growth factor receptor 2 inhibitor; HR, hormone receptor; mTORi, mammalian target of rapamycin inhibitor; NSAI, non-steroidal aromatase inhibitor; SAI, steroidal aromatase inhibitor; WHO, world health organization

### Treatment sequences

In total, 650 treatment lines were given in this patient population. The median number of total treatment lines received was three (range 1–11). Reasons for treatment discontinuation were progressive disease (*n* = 480, 74%), toxicity (*n* = 25, 4%), death (*n* = 24, 4%), sufficient response (*n* = 17, 3%), mixed or insufficient response (*n* = 13, 2%) and other reasons (*n* = 13, 2%). Reasons for treatment discontinuation specified per treatment line are depicted in Table 2.

**Table 2:** Reasons for treatment discontinuation for first, second and third-line

	First-line	Second-line	Third-line
	*N* = 202 (%)	*N* = 161 (%)	*N* = 105 (%)
Progressive disease	151 (75)	117 (73)	76 (72)
Sufficient response	9 (5)	4 (2)	–
Death	5 (2)	4 (2)	2 (2)
Toxicity	4 (2)	4 (2)	10 (10)
Mixed or insufficient response	3 (1)	2 (1)	2 (2)
Other reasons	3 (1)	8 (5)	2 (2)
Ongoing	27 (13)	22 (14)	13 (12)

Of the total patient population, 161 (80%) patients started second-line treatment within follow-up time. Most patients who did not move to second-line treatment did so because of ongoing treatment with first-line treatment at the cutoff date (*n* = 27, 13%). The other patients who did not move to second-line failed to do so due to death (*n* = 5, 2%), or loss to follow up (*n* = 5, 2%).

Of the patients starting first-line ET of any kind (*n* = 174), 135 (78%) started with any second-line therapy within follow-up time, which was ET in 104 (77%) patients. Twenty-seven (16%) patients were still ongoing on their first-line treatment until cutoff date, five (3%) patients died and five (3%) patients were lost to follow up. Seventy-nine patients (39%) were ongoing on their last treatment line at time of censoring or cutoff date. Of the patients who started with NSAI monotherapy (*n* = 91), 71 (78%) started with any second-line therapy within follow-up time. Fifteen (17%) patients were still ongoing on first-line treatment until cutoff date, 4 (4%) died, and 1 (1%) was lost to follow up.

**Fig. 2:**
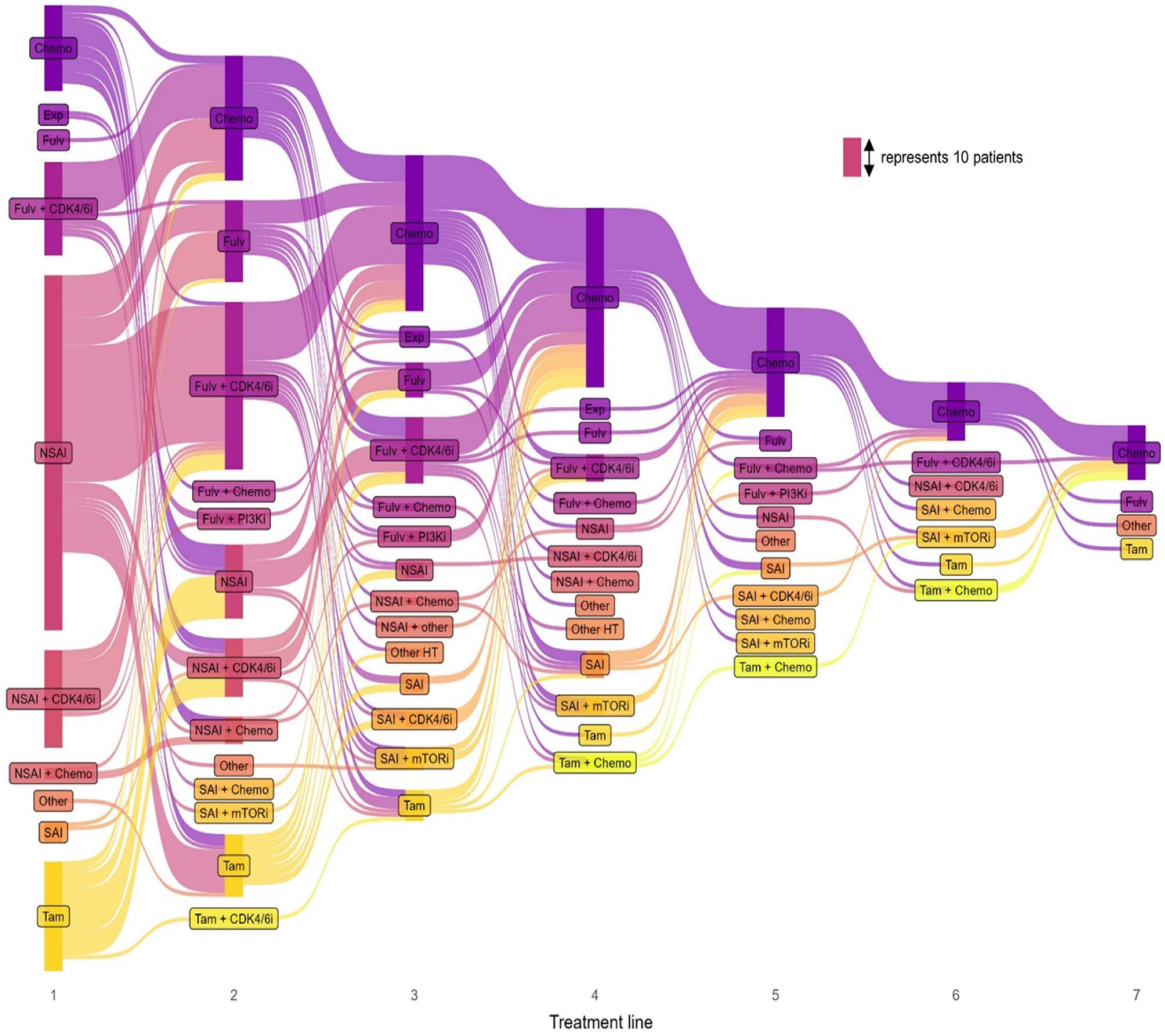
Sankey plot of treatment patterns in HR-positive HER2-negative patients up to the seventh treatment line. CDK4/6i, CDK4/6 inhibitor; Chemo, chemotherapy; Fulv, fulvestrant; HT, hormonal therapy; mTORi, mTOR inhibitor; NSAI, non-steroidal aromatase inhibitor; PI3Ki, PI3K inhibitor; SAI, steroidal aromatase inhibitor; Tam, tamoxifen. Other: chemotherapy + VEGF/PARP inhibitor, sacituzumab govitecan

All treatment sequences applied in the whole patient population are shown in Fig. 2. Patients who did not move to a next treatment line are visualized as such, irrespective of the reason why they did not move to the next line. The Sankey plot with treatment sequences including visualization of ongoing treatment, loss to follow up and death is shown in the supplementary files S1. Most patients started with ET, whereas 13% (*n* = 26) did not start with ET. All these patients started with chemotherapy (± endocrine or targeted therapies), because of visceral crisis or otherwise threatening symptomatic disease. In nine of these 26 (35%) patients, first-line chemotherapy was eventually discontinued due to sufficient response. Eighteen of the 26 (69%) patients starting with chemotherapy were treated with ET in later treatment lines. Treatment sequences in patients who started first-line therapy before and after reimbursement of CDK4/6i and the individual Sankey plots sorted by first-line treatment are shown in the supplementary files S2-8.

### Treatment duration

**Fig. 3:**
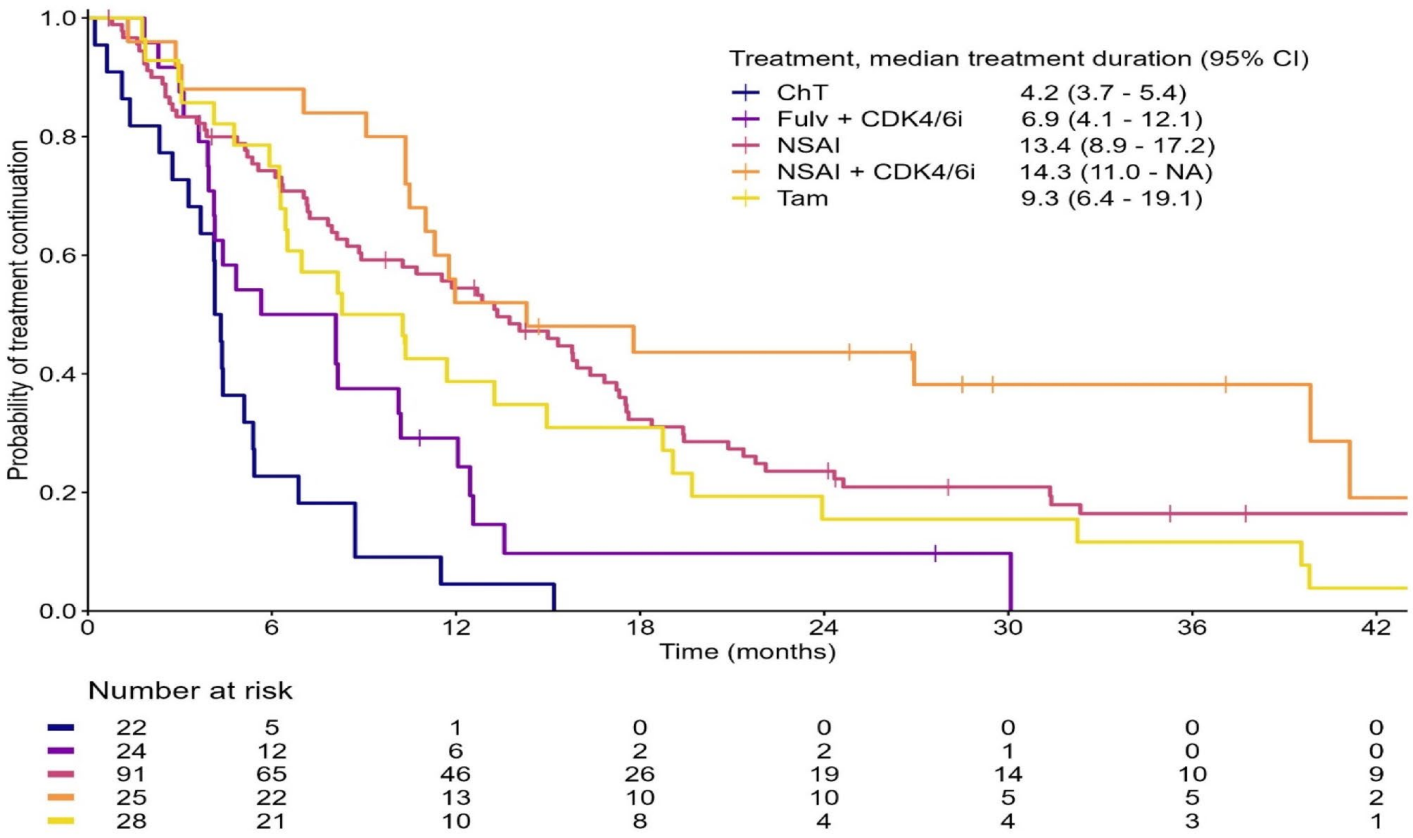
Kaplan Meier curve for treatment duration of first-line treatments given in HR-positive HER2-negative patients. Treatment options with ≤ 5 patients are not shown in this figure. *CI* confidence interval, *ChT* chemotherapy, *Fulv* fulvestrant, *CDK4/6i* CDK4/6 inhibitor, *NSAI* non-steroidal aromatase inhibitor, *Tam*tamoxifen

Median follow-up time was 36.8 months. The Kaplan–Meier curves with TTD on first-line treatment are shown in Fig. 3. Median TTD was 4.2 months (95% confidence interval (CI), 3.7–5.4), 6.9 months (95% CI 4.1–12.1), 13.4 months (95% CI 8.9–17.2), 14.3 months (95% CI 11.0–NA), 9.3 months (95% CI 6.4–19.1) for the chemotherapy, fulvestrant + CDK4/6i, NSAI, NSAI + CDK4/6i and tamoxifen group, respectively. In the chemotherapy group, 38% (*n* = 8) discontinued due to sufficient response. Median TTD was comparable in patients treated with NSAIs and NSAIs + CDK4/6i. A statistically significant difference was observed between the chemotherapy and fulvestrant + CDK4/6i group versus the NSAI group with *p*-values of < 0.001 and 0.004, respectively.

Median TTD for any type of first-line ET was 11.5 months (95% CI 9.07–13.6). Median TTD for any type of second-line ET in patients who were also treated with first-line ET, was 6.1 months (95% CI 4.96–8.54). Median TTD for any type of third-line ET in patients who only received first and second-line treatment with ET was 4.37 (95% CI 3.35–7.03). Median TTD for any type of first ET started after a non-ET in the treatment sequence was 3.5 months (95% CI 2.53–4.53). Median TTD on everolimus + exemestane (*n* = 18) in any treatment line (2–9) was 3.22 months (95% CI 1.97–11.9).

**Fig. 4:**
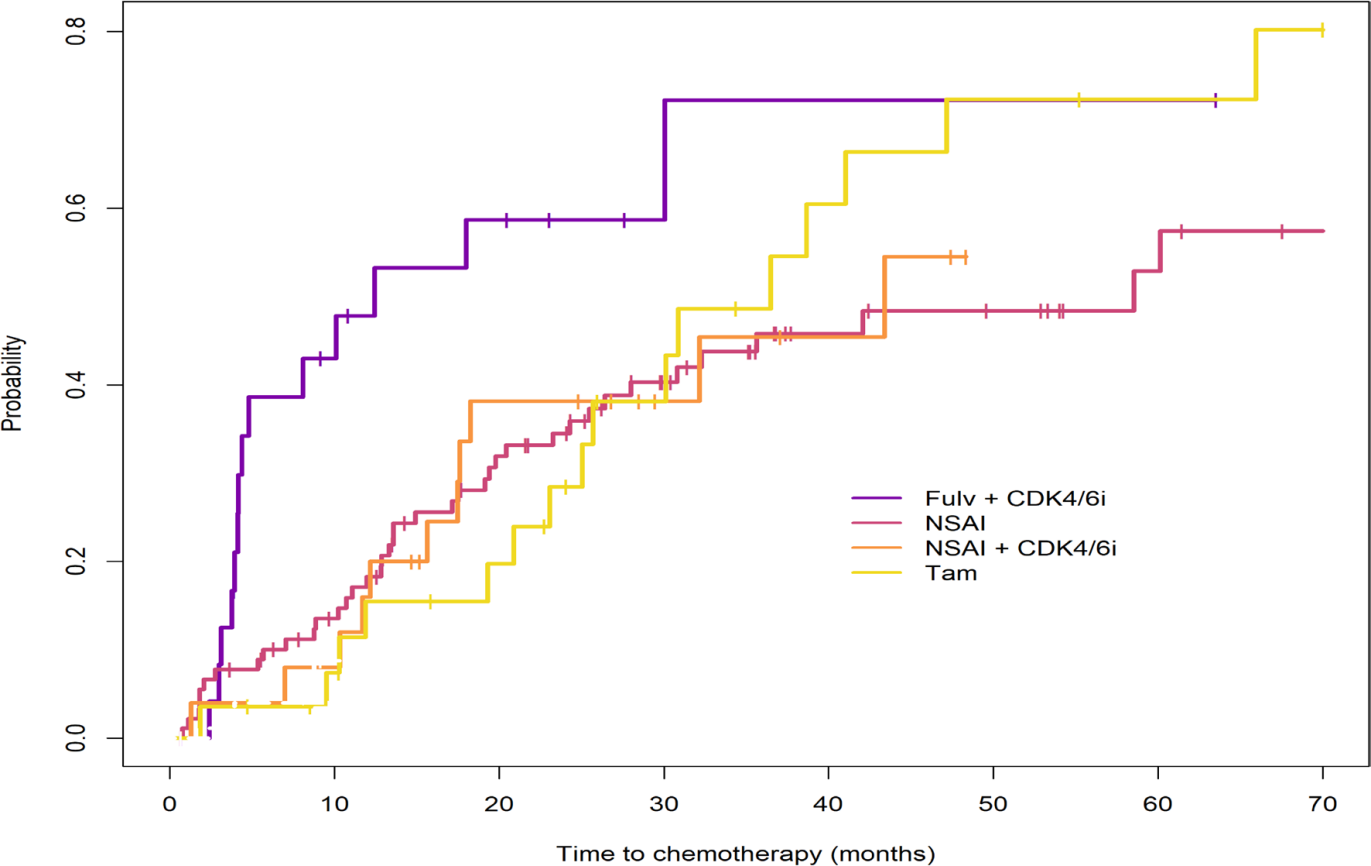
Cumulative incidence for time to chemotherapy (TTC) stratified by first-line systemic treatment. Death was taken as competing risk, but not plotted in this figure to improve readability. Full figure available in S9. Fulv, fulvestrant; CDK4/6i, CDK4/6 inhibitor; NSAI, non-steroidal aromatase inhibitor; Tam, tamoxifen; TTC, time to chemotherapy

Figure 4 shows the cumulative incidence of start of chemotherapy for those patients who started with ET (*n* = 168), stratified by first-line systemic treatment, using a competing risk approach. Of these patients, 82 started chemotherapy within follow-up time and 21 patients died before receiving chemotherapy. Median TTC was longest in patients treated with first-line NSAI and shortest in patients treated with first-line fulvestrant + CDK4/6i. Median TTC was 12.5 months (95% CI 4.4–NA), 58.5 months (95% CI 30.8–95.4), 43.4 months (95% CI 18.3–NA) and 36.5 months (95% CI 25.7–NA) for fulvestrant + CDK4/6i, NSAI, NSAI + CDK4/6i and tamoxifen, respectively. No statistically significant differences were observed in the cumulative incidence curves in comparison with the NSAI group. Figure S9 shows TTC with death plotted as competing risk.

**Fig. 5:**
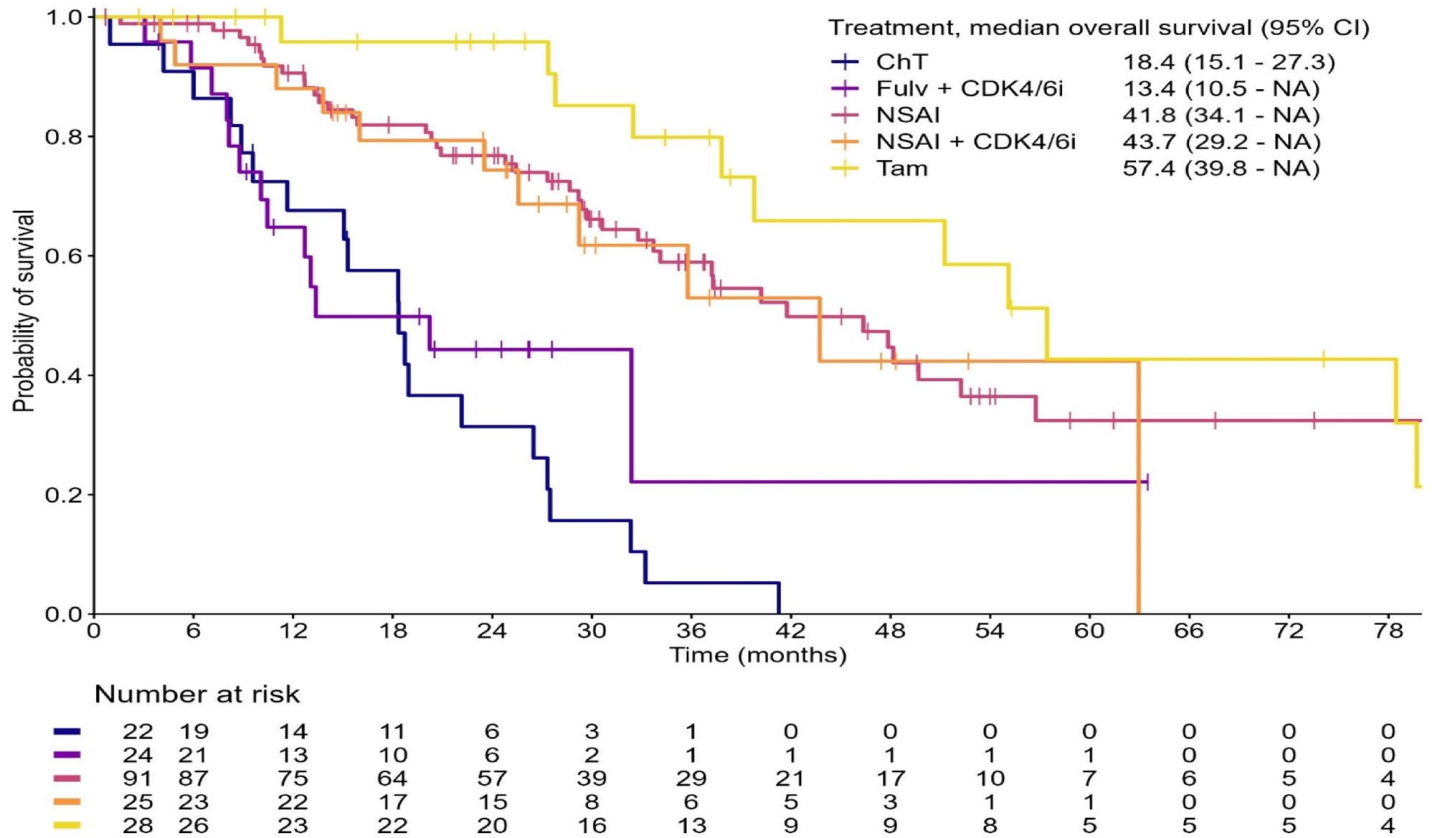
Kaplan Meier curve for overall survival stratified by first-line treatments given in HR-positive HER2-negative patients. Treatment options with ≤ 5 patients are not shown in this figure. CI, confidence interval; ChT, chemotherapy; Fulv, fulvestrant; CDK4/6i, CDK4/6 inhibitor; NSAI, non-steroidal aromatase inhibitor; Tam, tamoxifen

Median OS for the whole patient population was 37.2 months (95% CI 32.3–47.8). OS of patients stratified by first-line treatment are shown in Fig. 5. Median OS was 18.4 months (95% CI 15.1–27.3), 13.4 months (95% CI 10.5–NA), 41.8 months (95% CI 34.1–NA), 43.7 months (95% CI 29.2–NA) and 57.4 months (95% CI 39.8–NA) for chemotherapy, fulvestrant + CDK4/6i, NSAI, NSAI + CDK4/6i and tamoxifen group, respectively. A statistically significant difference was observed between the fulvestrant + CDK4/6i and chemotherapy group versus the NSAI group, with *p*-values of 0.002 and < 0.001, respectively.

Median time from discontinuation of the last therapy line until death varied widely (range 0–364 days), with a median of 14 days (interquartile range, 0.5–31.5). When no explicit information was present in the electronic medical record about discontinuation of treatment, treatment until death was assumed.

## Discussion

In this study, we presented an overview of the real-world applied treatment sequences at a comprehensive cancer center for HR + HER2- ABC patients and we determined the survival outcomes of patients treated with different treatment sequences. We observed that a large variety of different treatment sequences is applied in clinical practice. The number of treatment lines (1–11) and the unique sequences (> 20 unique patterns in the first two lines only) varied greatly.

In the current ESMO Clinical Practice guidelines, ET combined with CDK4/6i is the recommended first-line treatment [12]. No significant differences were observed in OS in our study between patients treated with first-line NSAI or NSAI + CDK4/6i, which was also the case in the study of *Schneeweiss *et al. [13]. When comparing baseline characteristics of both groups, patients treated with NSAI + CDK4/6i as first-line treatment tended to be more often endocrine resistant and had more often visceral disease, which may have influenced the choice for combination therapy.

Patients receiving fulvestrant + CDK4/6i as first-line treatment showed shorter TTD and TTC. This short TTD is consistent with recent PFS data of patients on first-line fulvestrant + CDK4/6i in the placebo arm of the INOVA-120 trial [14]. Those who started on fulvestrant + CDK4/6i had significantly shorter OS than the reference group. This is to be expected, as fulvestrant + CDK4/6i in first-line is only given when the patient has progressed during or < 1 year after treatment with an AI in adjuvant setting [12].

Median TTD of patients on first-line chemotherapy was only 4.2 months and was comparable when excluding the patients who stopped chemotherapy due to sufficient response. OS for patients who started with chemotherapy was significantly worse than those of the reference group. This is unsurprising, given the fact that all these patients had threatening symptomatic disease, and OS has been shown to be around 19 months when chemotherapy is given upfront in literature [15].

Patients who started first-line treatment on tamoxifen showed non-significantly shorter TTD and TTC compared to those who started on NSAI. However, OS of patients on tamoxifen was considerably longer than the OS of all other groups, although this difference did not reach statistical significance. Patients treated with first-line tamoxifen tended to be younger, which might have contributed to their longer OS. As median TTC was not longer in the tamoxifen group compared to the NSAI group, ‘stacking all available ET’ does not seem to be the primary reason for longer OS in this group. Apparently, clinicians are well equipped to select patients with a favorable prognosis, and chose to treat them with tamoxifen first. We are well aware that decision making of clinicians is based on a combination of scientific evidence, analytic thinking and recognition of complex patterns, and possibly also on other influences, such as experience with a drug and marketing strategies of the pharmaceutical industry. Prior research has shown that ‘the gut feeling’ of a clinician is actually the use of the recognition of complex patterns by experience [16]. These patterns may not be modelable using only the data documented in the patient files. Our hypothesis is that the experience of the clinicians enables them to correctly distinguish patients in need for a fast response, in whom a more toxic first-line treatment is warranted, from patients with a normal or favorable prognosis, in whom endocrine (mono)therapy with low toxicity profiles could be applied first. Fear that patients may never get around to treatment with CDK4/6i altogether when they do not get CDK4/6i in first-line treatment was not supported by our study results, as only 3% of patients who started NSAI monotherapy did not live to get treatment in second-line therapy.

In our study population mOS was 37.2 months. This is lower than reported in a large French real-world cohort of HR + HER2- ABC patients under 70 years old with mOS of 42.3 months and 46.9 months for patients < 40 years and between 40 and 69 years, respectively. This might be explained by a different casemix, as our center is a tertiary referral center. When compared to mOS in phase III trials on CDK4/6i in first-line treatment, mOS is significantly less than mOS reported in the arms of PALOMA-2 (51.2 and 53.9 months, respectively), PARSIFAL-LONG (68.5 and 61.9 months, respectively), and SONIA (45.9 and 53.7 months, respectively) [17]. These significant differences might be explained by strict selection of patients in these randomized trials.

One of the strengths of this study is the comprehensive overview of treatment sequences in HR + HER2- ABC patients in clinical practice. As patient characteristics influenced the choice of first-line treatment, survival outcomes between groups should not be directly compared to one another due to confounding by indication. This, for example, is the case for the limited number of patients who received tamoxifen or chemotherapy as first-line treatment. There was a limited number of patients receiving any specific order of first, second, and third-line treatment. Consequently, it was not possible to compare the total treatment duration between different consecutive treatment orders.

Future research regarding treatment sequences should be performed. For earlier treatment lines, this can be done by performing head-to-head randomized clinical trials, whereas for later treatment lines matched cohort studies are probably more feasible. However, as median TTDs shorten in later endocrine treatment lines as compared to earlier treatment lines in our study, it seems reasonable to base decision making after third-line on the response of individual patients, rather than setting up matched cohort studies, with all the methodological challenges tied to such a design. It may be useful to investigate the role of tamoxifen in ABC treatment further, as patients treated with first-line tamoxifen in our study did seem to do better than patients starting on NSAI with or without CDK4/6i.

## Conclusions

In this real-world cohort, we observed a wide variety of different treatment sequences applied in daily clinical practice, some of which discordant with current guidelines. Patients treated with first-line tamoxifen showed a non-significantly longer OS, although survival outcomes were confounded by indication. Fear that patients who do not get treatment with CDK4/6i in first-line may never get around to treatment with CDK4/6i altogether was not supported by our study results.

## Supplementary Information

Below is the link to the electronic supplementary material.

Supplementary file1 (DOCX 3347 KB)

## Data Availability

The dataset generated during the current study is available from the corresponding author on reasonable request.
