# Supplementary material for: Treatment sequences and survival outcomes in advanced HR + HER2- breast cancer patients: a real-world cohort
Source: Breast Cancer Res Treat. 2024 Nov 7;210(1):115–24. doi: 10.1007/s10549-024-07542-0 (PMC11787233; doi:10.1007/s10549-024-07542-0)

**Fig. S1** Sankey plot of treatment patterns in patients with HR+ HER2- ABC, including reasons not to move to a next therapy line.
CDK4/6i, CDK4/6 inhibitor; Chemo, chemotherapy; Fulv, fulvestrant; Exp, experimental therapy; HT, hormonal therapy; LTFU, loss to follow up; mTORi, TOR inhibitor; NSAI, non-steroidal aromatase inhibitor; PI3Ki, PI3K inhibitor; SAI, steroidal aromatase inhibitor; Tam, tamoxifen.

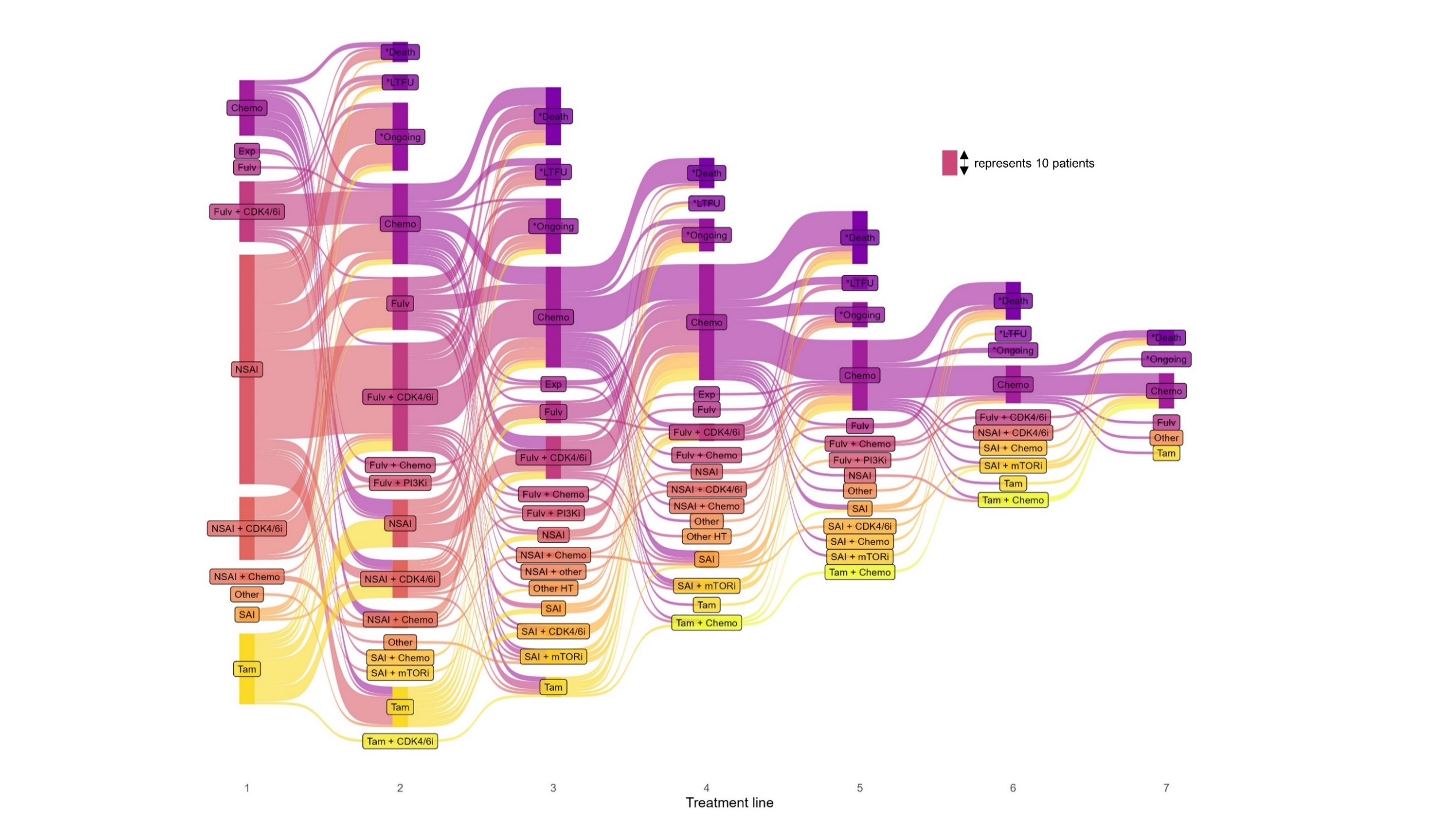


**Fig. S2** Sankey plot of treatment patters in HR+ HER2- patients who started first-line treatment before reimbursement of CDK4/6 inhibitors (August 1, 2017), n = 49.
CDK4/6i, CDK4/6 inhibitor; Chemo, chemotherapy; Exp, experimental therapy; Fulv, fulvestrant; HT, hormonal therapy; mTORi, mTOR inhibitor; NSAI, non-steroidal aromatase inhibitor; PI3Ki, PI3K inhibitor; SAI, steroidal aromatase inhibitor; Tam, tamoxifen.

***
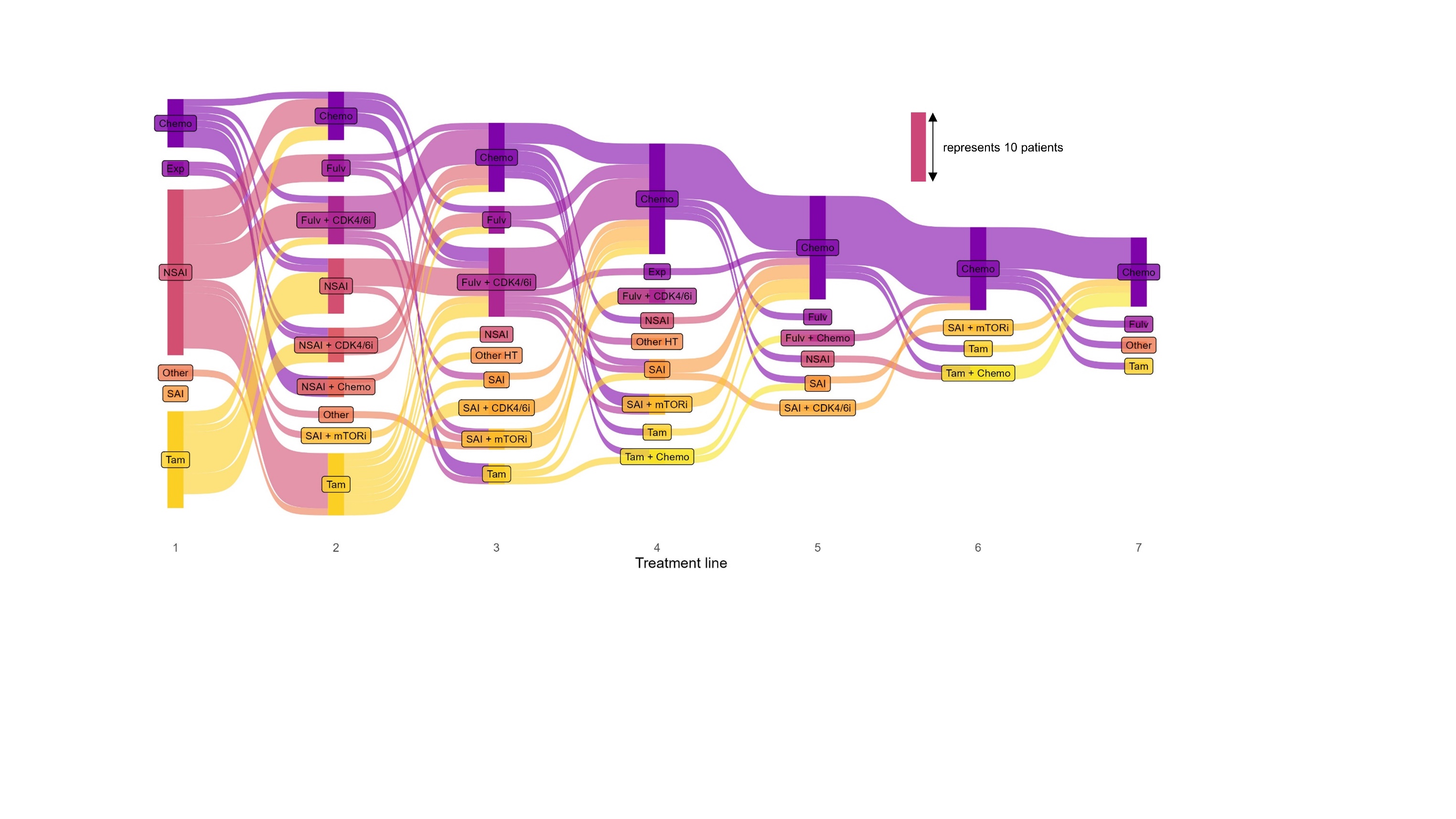
***

**Fig. S3** Sankey plot of treatment patters in HR+ HER2- patients who started first-line treatment after reimbursement of CDK4/6 inhibitors (August 1, 2017), n = 153.
CDK4/6i, CDK4/6 inhibitor; Chemo, chemotherapy; Exp, experimental therapy; Fulv, fulvestrant; HT, hormonal therapy; mTORi, mTOR inhibitor; NSAI, non-steroidal aromatase inhibitor; PI3Ki, PI3K inhibitor; SAI, steroidal aromatase inhibitor; Tam, tamoxifen.

**
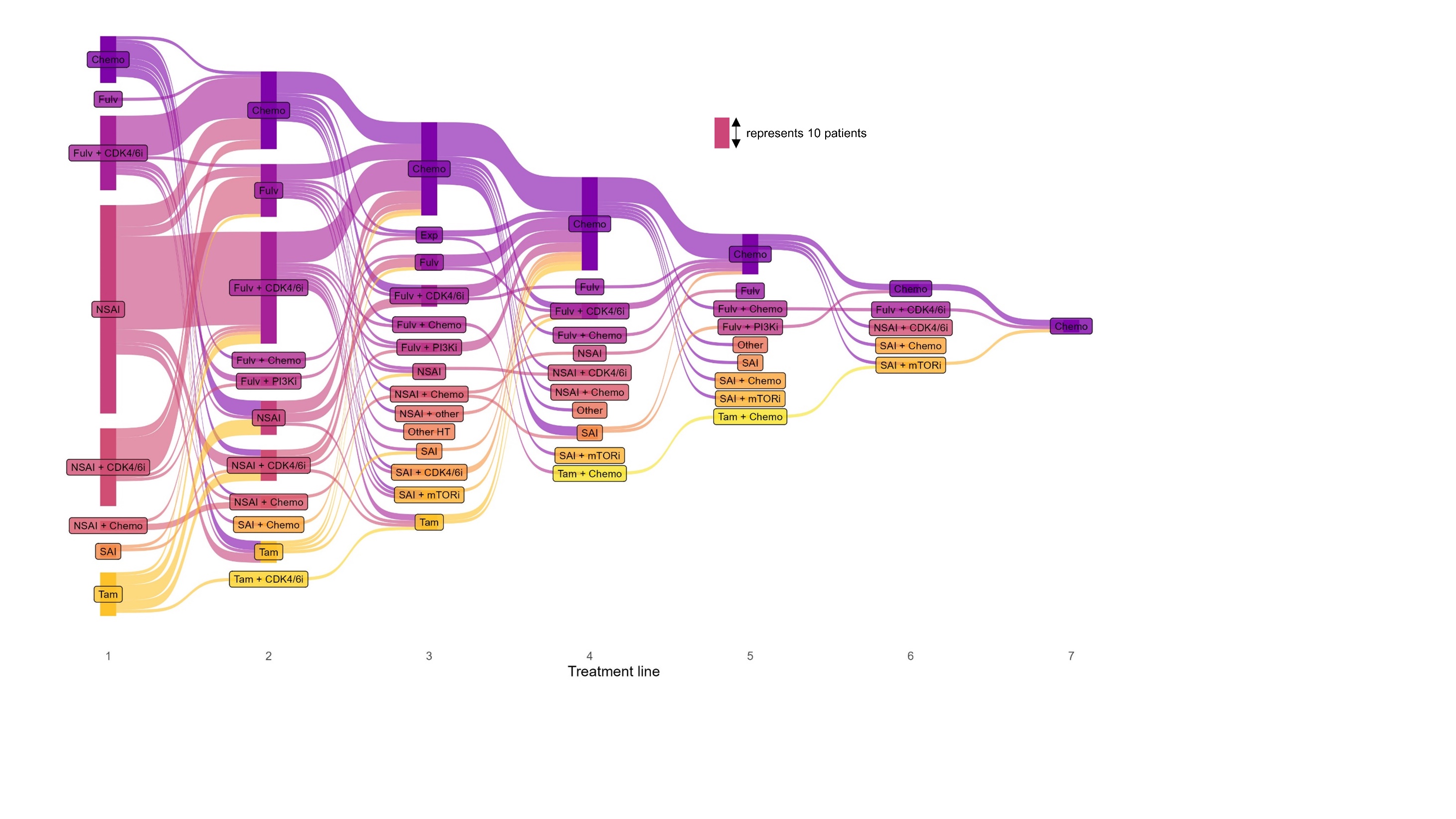
**

**Fig. S4** Sankey plot of treatment patterns in patients with HR+ HER2- ABC treated with NSAI monotherapy in first-line, n = 91.
CDK4/6i, CDK4/6 inhibitor; Chemo, chemotherapy; Exp, experimental therapy; Fulv, fulvestrant; HT, hormonal therapy; LTFU, loss to follow up; mTORi, mTOR inhibitor; NSAI, non-steroidal aromatase inhibitor; PARPi, PARP inhibitor; PI3Ki, PI3K inhibitor; SAI, steroidal aromatase inhibitor; Tam, tamoxifen.

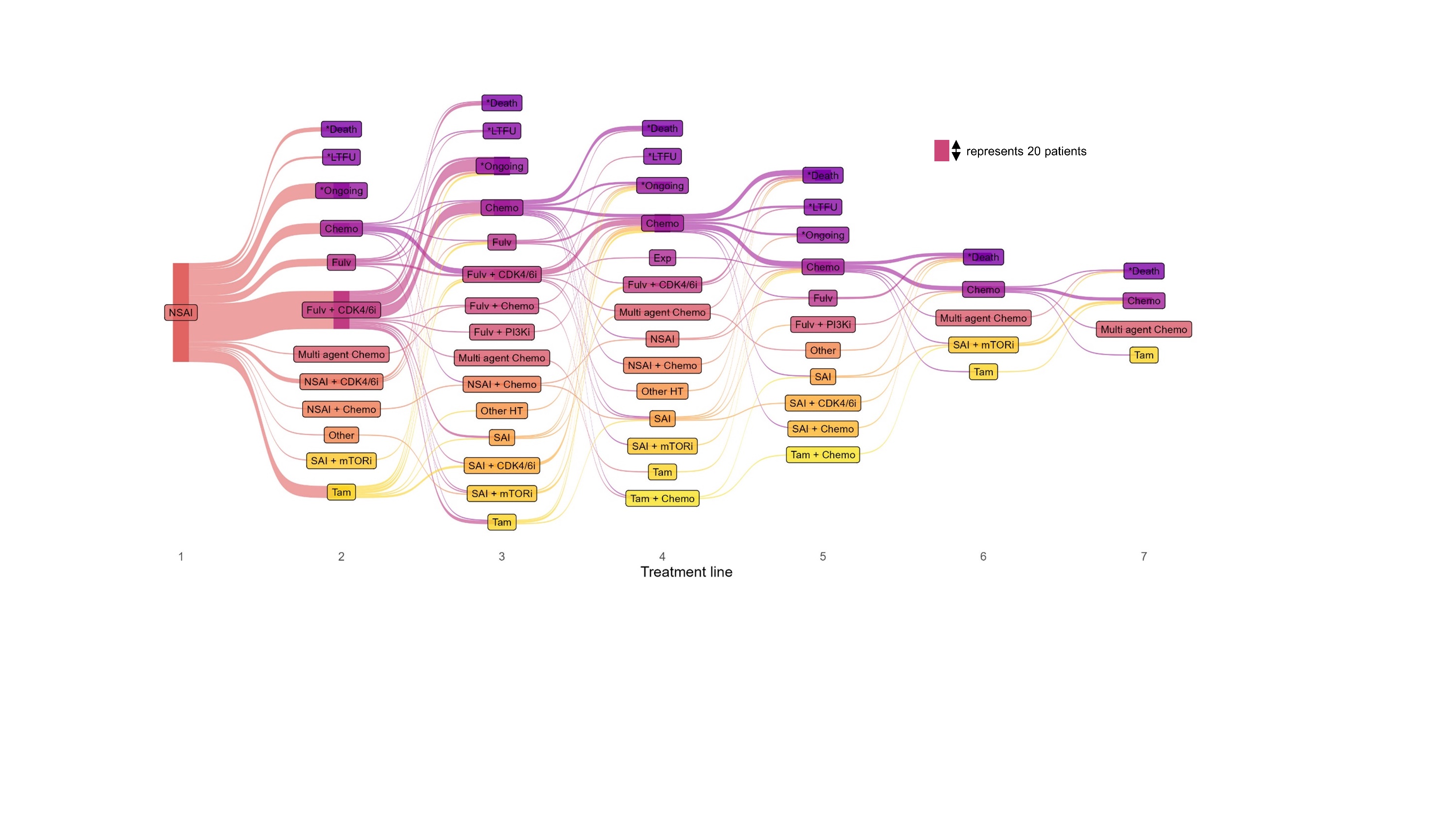


**Fig. S5** Sankey plot of treatment patterns in patients with HR+ HER2- ABC treated with NSAI + CDK4/6i in first-line, n = 25.
CDK4/6i, CDK4/6 inhibitor; Chemo, chemotherapy; Exp, experimental therapy; Fulv, fulvestrant; LTFU, loss to follow up; mTORi, mTOR inhibitor; NSAI, non-steroidal aromatase inhibitor; PI3Ki, PI3K inhibitor; SAI, steroidal aromatase inhibitor.

***
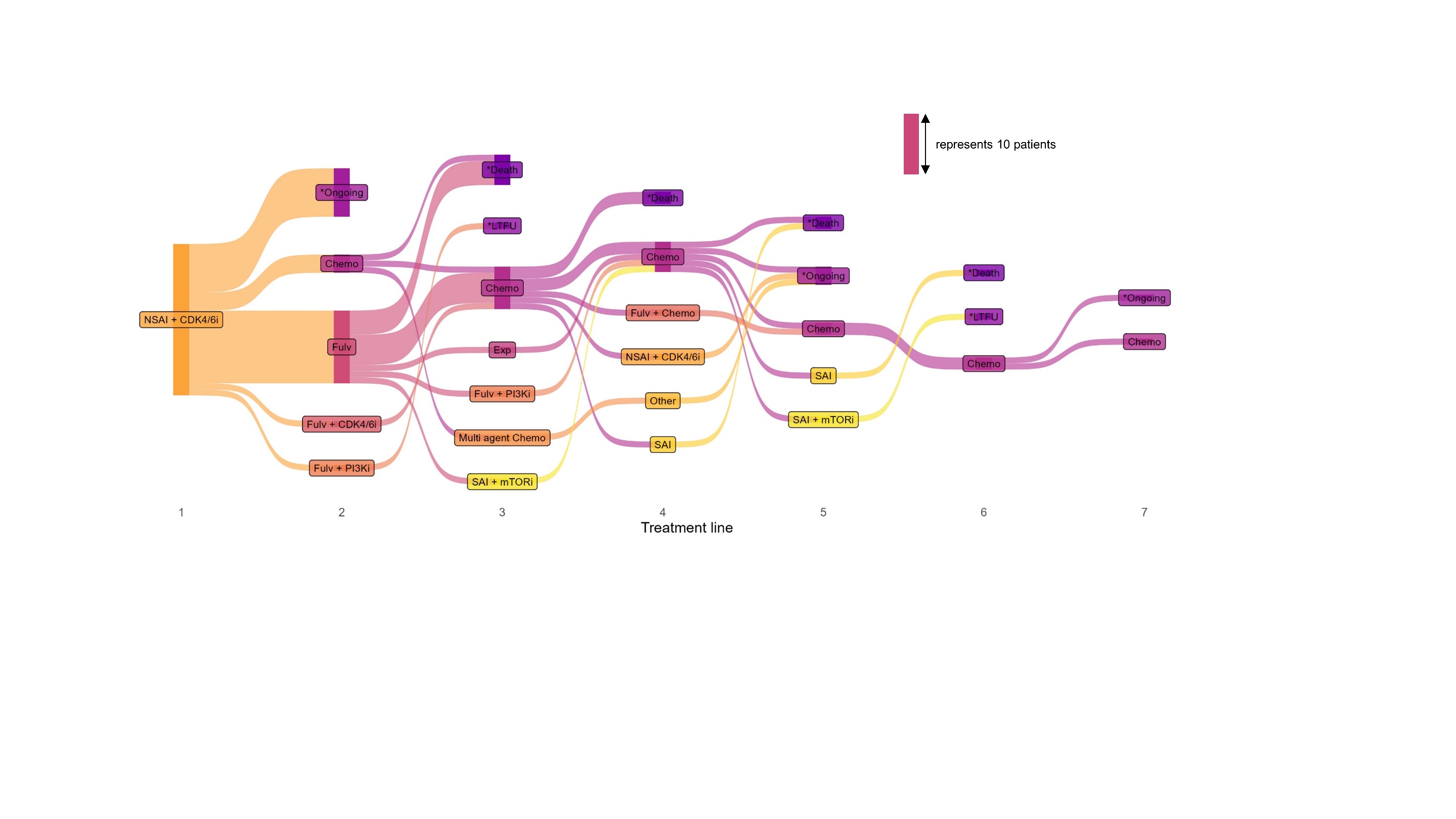
***

**Fig. S6** Sankey plot of treatment patterns in patients with HR+ HER2- ABC treated with tamoxifen monotherapy in first-line, n = 28.
CDK4/6i, CDK4/6 inhibitor; Chemo, chemotherapy; Fulv, fulvestrant; HT, hormonal therapy; LTFU, loss to follow up; mTORi, mTOR inhibitor; NSAI, non-steroidal aromatase inhibitor; PI3Ki, PI3K inhibitor; SAI, steroidal aromatase inhibitor; Tam, tamoxifen.


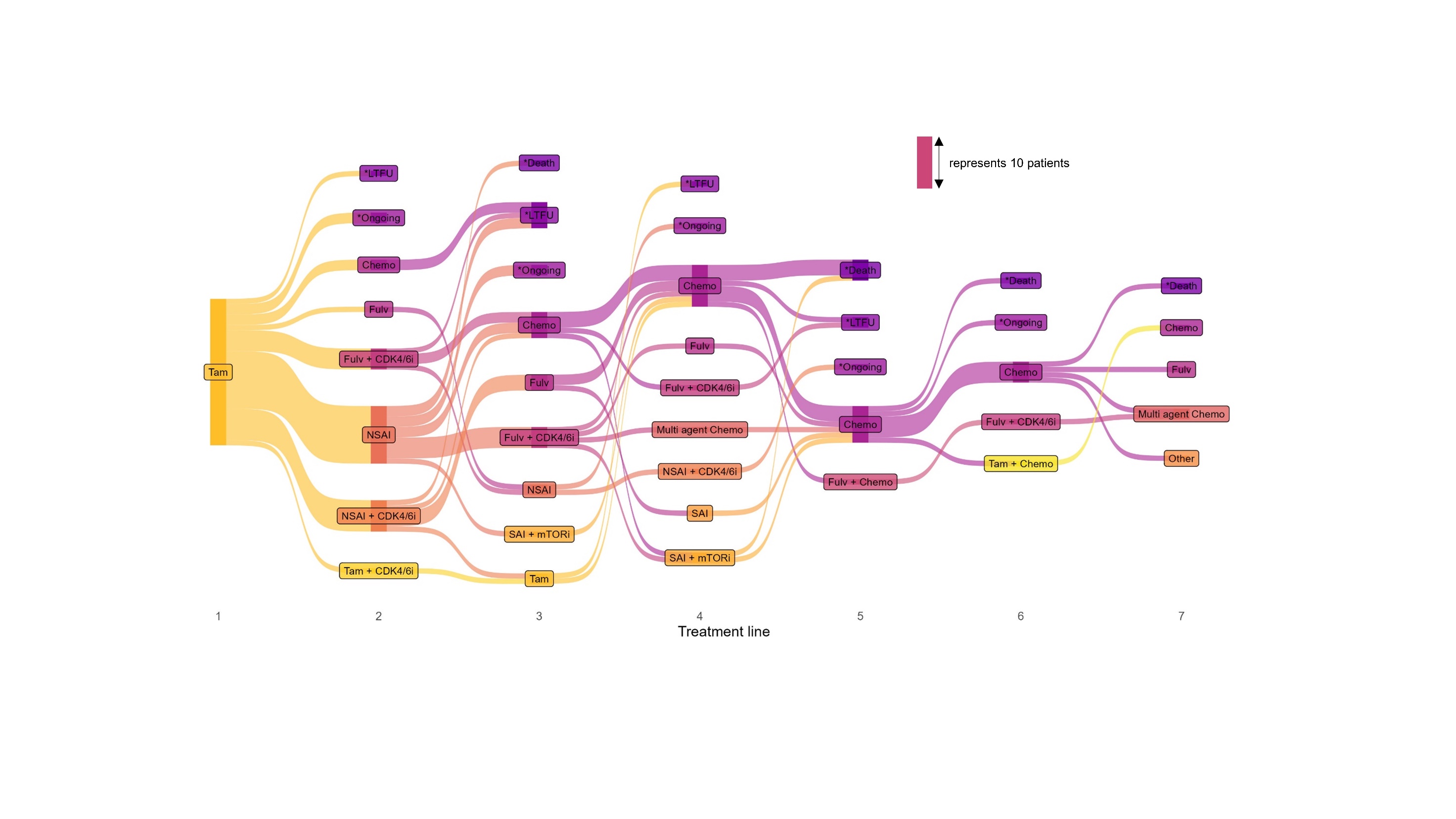


**Fig. S7** Sankey plot of treatment patterns in patients with HR+ HER2- ABC treated with fulvestrant + CDK4/6i in first-line, n = 24.
CDK4/6i, CDK4/6 inhibitor; ChT, chemotherapy; Fulv, fulvestrant; Exp, experimental therapy; HT, hormonal therapy; LTFU, loss to follow up; mTORi, mTOR inhibitor; NSAI, non-steroidal aromatase inhibitor; PI3Ki, PI3K inhibitor; SAI, steroidal aromatase inhibitor.

**
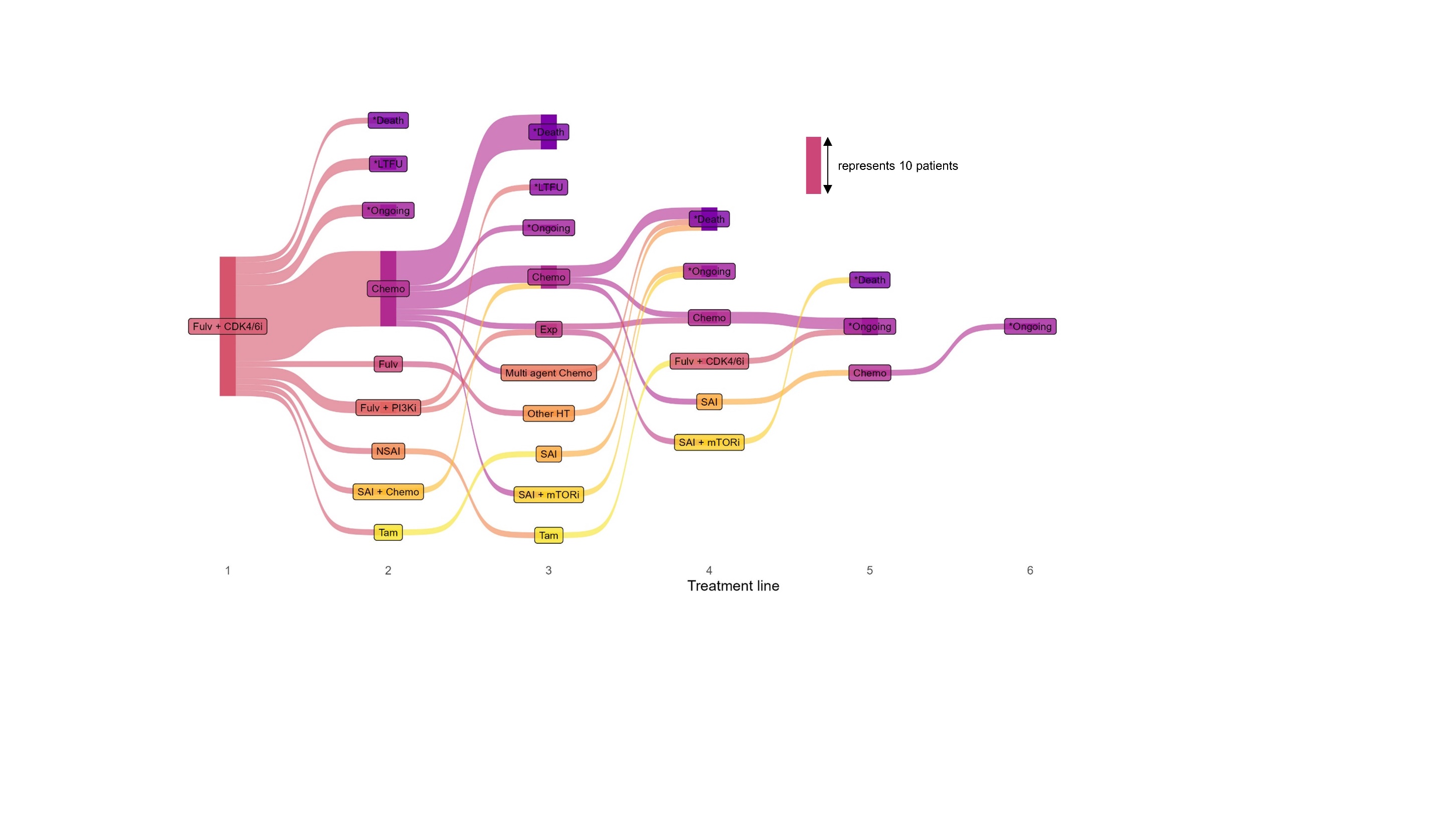
**

**Fig. S8** Sankey plot of treatment patterns in patients with HR+ HER2- ABC treated with chemotherapy (with or without ET/VEGFi) in first-line, n = 26.
CDK4/6i, CDK4/6 inhibitor; ChT, chemotherapy; ET, endocrine therapy; Fulv, fulvestrant; LTFU, loss to follow up; mTORi, mTOR inhibitor; NSAI, non-steroidal aromatase inhibitor; PI3Ki, PI3K inhibitor; SAI, steroidal aromatase inhibitor; VEGFi, VEGF inhibitor.


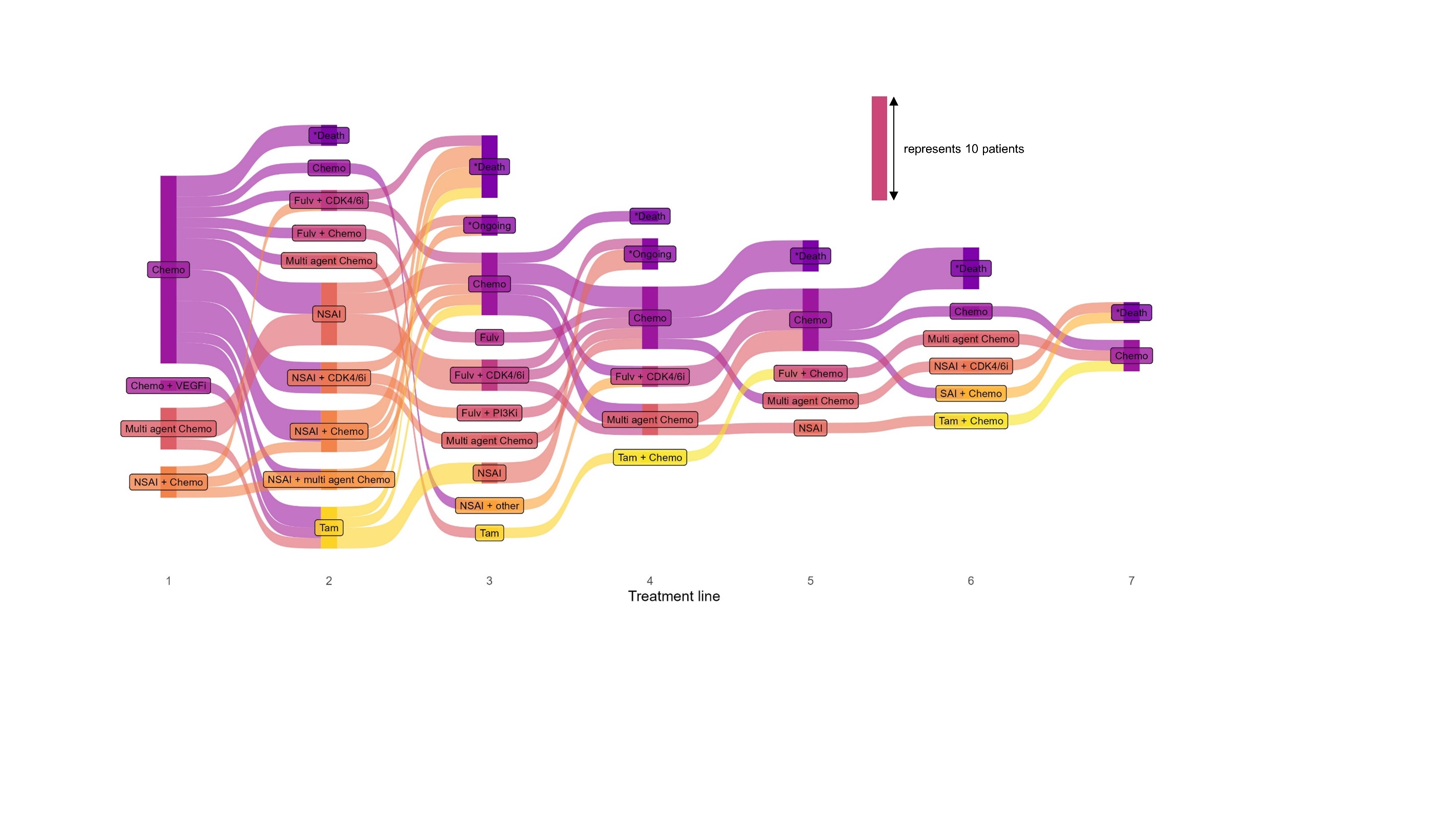


**Fig. S9** Cumulative incidence for time to chemotherapy (TTC) stratified by first-line systemic treatment. Death taken as competing risk, and plotted in this figure. Fulv, fulvestrant; CDK4/6i, CDK4/6 inhibitor; NSAI, non-steroidal aromatase inhibitor; Tam, tamoxifen; TTC, time to chemotherapy.

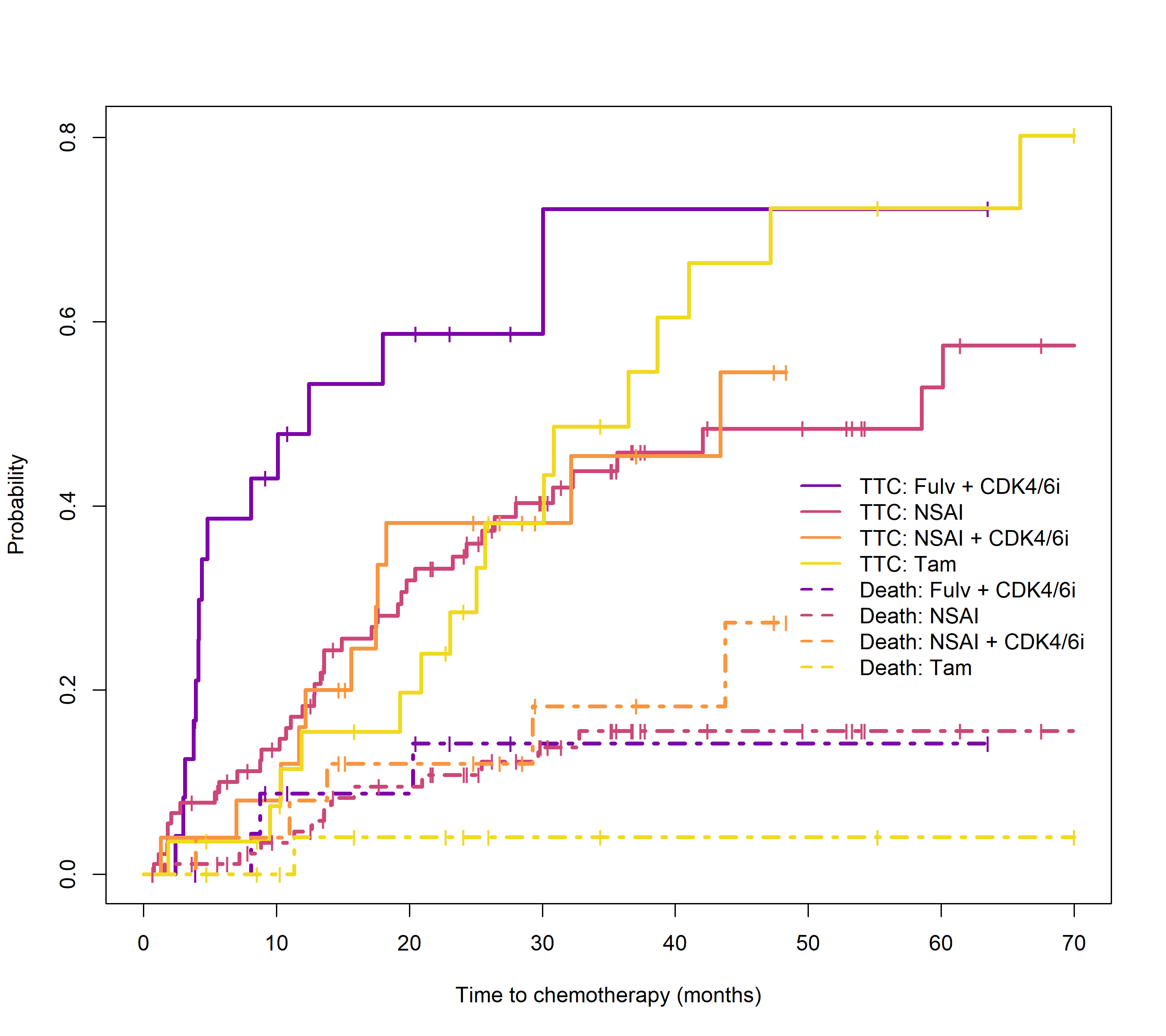

Supplement: Supplementary file 1 — Supplementary file1 (DOCX 3347 KB) [file 10549_2024_7542_MOESM1_ESM.docx]
